# Hypothesis on* Serenoa repens* (Bartram) small extract inhibition of prostatic 5*α*-reductase through an *in silico* approach on 5*β*-reductase x-ray structure

**DOI:** 10.7717/peerj.2698

**Published:** 2016-11-22

**Authors:** Paolo Governa, Daniela Giachetti, Marco Biagi, Fabrizio Manetti, Luca De Vico

**Affiliations:** 1 Department of Physical Sciences, Earth and Environment, University of Siena, Siena, Italy; 2 Department of Chemistry, University of Copenhagen, Copenhagen, Denmark; 3 Department of Biotechnology, Chemistry and Pharmacy, University of Siena, Siena, Italy

**Keywords:** *Serenoa repens* (Bartram) Small, Benign prostatic hyperplasia, 5α-reductase, Molecular docking, PyRosetta, AutoDock

## Abstract

Benign prostatic hyperplasia is a common disease in men aged over 50 years old, with an incidence increasing to more than 80% over the age of 70, that is increasingly going to attract pharmaceutical interest. Within conventional therapies, such as *α*-adrenoreceptor antagonists and 5*α*-reductase inhibitor, there is a large requirement for treatments with less adverse events on, e.g., blood pressure and sexual function: phytotherapy may be the right way to fill this need. *Serenoa repens* standardized extract has been widely studied and its ability to reduce lower urinary tract symptoms related to benign prostatic hyperplasia is comprehensively described in literature. An innovative investigation on the mechanism of inhibition of 5*α*-reductase by *Serenoa repens* extract active principles is proposed in this work through computational methods, performing molecular docking simulations on the crystal structure of human liver 5*β*-reductase. The results confirm that both sterols and fatty acids can play a role in the inhibition of the enzyme, thus, suggesting a competitive mechanism of inhibition. This work proposes a further confirmation for the rational use of herbal products in the management of benign prostatic hyperplasia, and suggests computational methods as an innovative, low cost, and non-invasive process for the study of phytocomplex activity toward proteic targets.

## Introduction

Benign prostatic hyperplasia (BPH) is a non-cancerous growth of the prostatic gland, due to hyper-proliferation of both stromal and glandular prostatic elements (Shrivastava & Gupta, 2012).

With increasing life expectancy, BPH incidence is in continuous growth; it has been estimated that the annual cost of managing patients with BPH overcomes $4 billion (Shrivastava & Gupta, 2012). Pharmacological interest in BPH is therefore likely to rise in the coming years.

About 60% of male population aged over 50 years shows histological symptoms of BPH, and this grows to 80% over the age of 70 (Aggarwal et al., 2012). BPH is a chronic and progressive disease, with a multifactorial etiology related, among others, to androgenic activity in prostatic tissue (Kumar, Malla & Kumar, 2013). An increase in levels of dihydrotestosterone, the most potent androgen in our organism, is particularly observed in BPH tissue (Carson & Rittmaster, 2003). The main clinical manifestation of BPH affects the lower urinary tract, and can be divided in irritative and obstructive symptoms.

**Figure 1:**
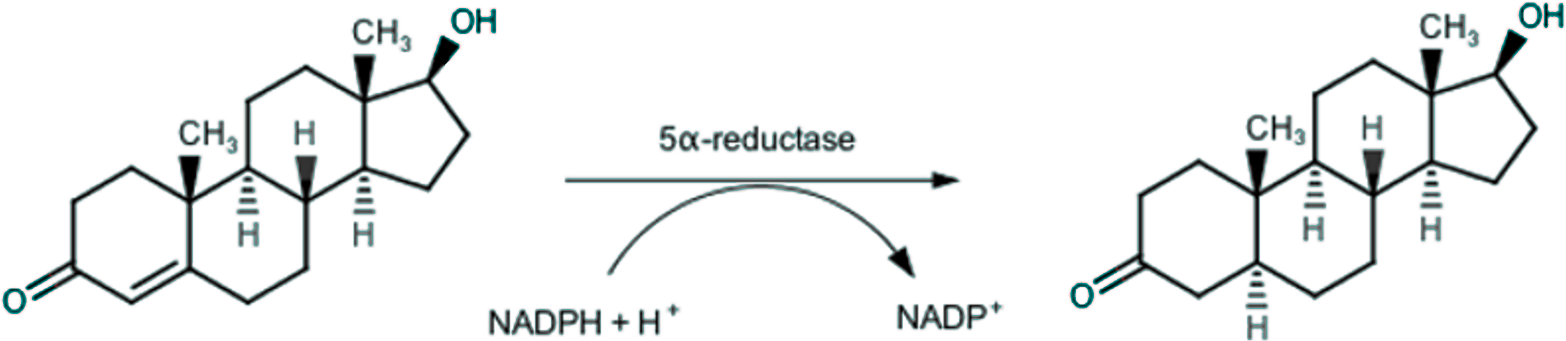
Conversion of testosterone into 5*α*-dihydrotestosteone by 5*α*-reductase.

Endogenous androgens, such as testosterone and dihydrotestosterone, play a key-role in growth and development of prostatic tissue, and thus also in prostatic diseases, especially in BPH. In the prostatic gland, testosterone is irreversibly converted through a 5*α*-reductase (5AR) catalyzed reaction (Russell & Wilson, 1994), as depicted in Fig. 1, in the more active dihydrotestosterone, which is responsible for BPH (Rittmaster, 2008).

**Figure 2:**
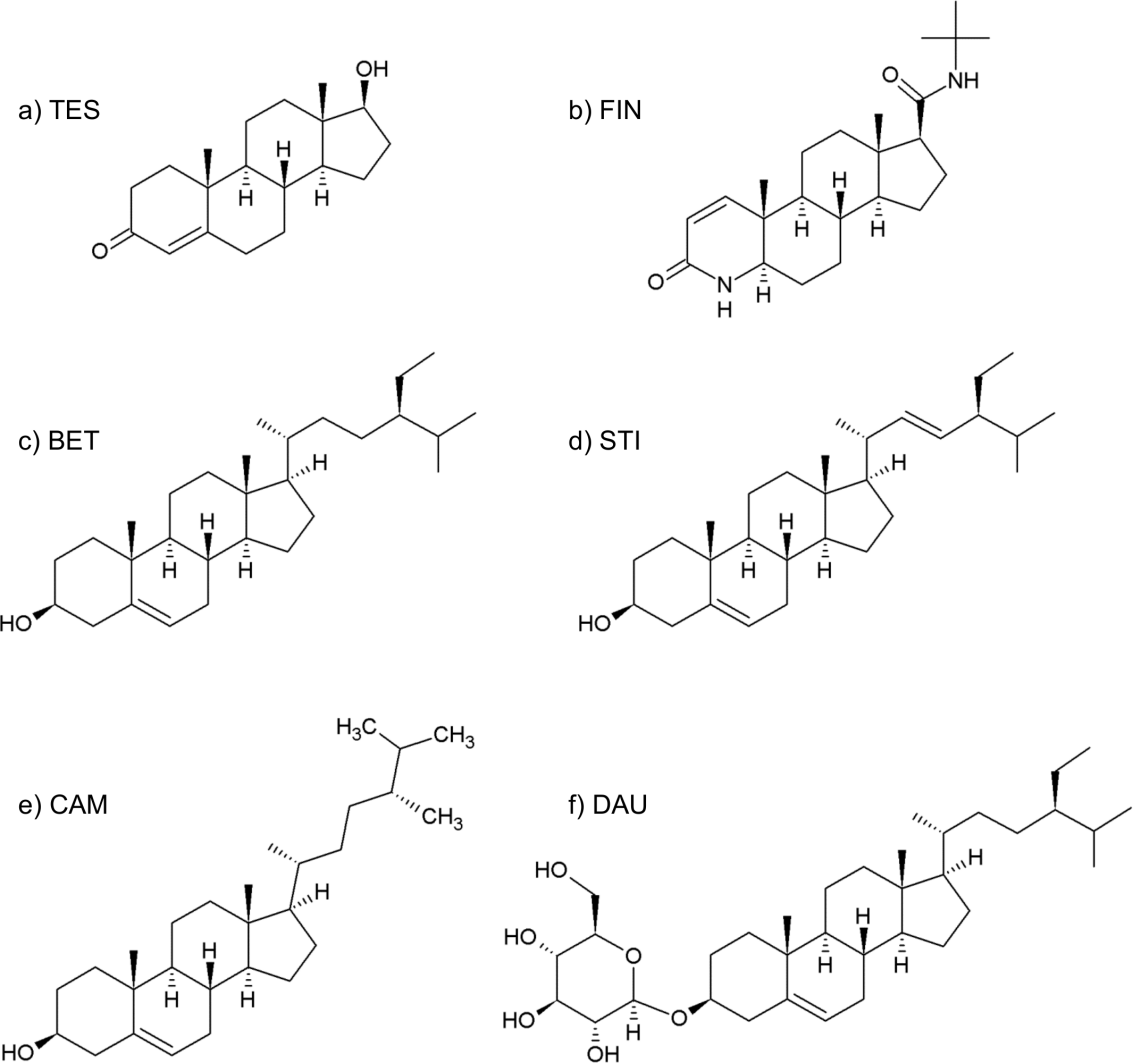
Formulas of (A) testosterone, (B) finasteride, (C) *β*-sitosterol, (D) stigmasterol, (E) campesterol, (F) daucosterol.

The management of BPH foresees various possibilities, including watchful-waiting, surgery and pharmacological therapies. The pharmacological approach mainly comprises *α*-adrenoceptor blockers, such as alfuzosin and tamsulosin (Fine & Ginsberg, 2008), and 5AR inhibitors, such as finasteride (see Fig. 2B) (Nickel et al., 2011).

Alongside other therapeutic approaches, phytoterapeutic agents, such as *Serenoa repens* (Bartram) Small extract (SRE), are also often prescribed for BPH treatment. A large number of papers established the clinical effectiveness of *Serenoa* in controlling lower urinary tract symptoms (LUTS) related to BPH (Champault, Patel & Bonnard, 1984; Carraro et al., 1996). A recent review stated SRE is as effective as conventional therapies in treating BPH related symptoms (Allkanjari & Vitalone, 2015). In clinical trials reported to date, side effects due to treatment with SRE are less frequent and severe than those observed with finasteride, primarily a lower incidence on sexual and gastro-enteric functions (Wilt et al., 1998). In 2012, a systematic review by the Cochrane group stated that no improvement in BPH related symptoms are provided by the use of SRE, compared to placebo (Tacklind et al., 2012). Nevertheless, this review included clinical trials performed by administering different SRE preparations, at different dosages. Bio equivalence of different herbal preparations is an everlasting issue, as the quality of the final product can be influenced by many manufacturing steps, such as the botanical source, the employed part of the plant, extraction process, used solvents and drug extract ratio. In order to reduce the results variability during clinical trials only standardized, chemically reproducible, extracts should be administered.

SRE mechanism of action remains unclear. Different mechanisms have been proposed, including anti-androgenic actions (Carilla et al., 1984), inhibition of 5AR (Bayne et al., 1999), anti-inflammatory (Breu et al., 1992; Vela-Navarrete et al., 2002), anti-aedematous and anti-oxidant effects (Tarayre et al., 1983), and antiproliferative influence leading to apoptosis through the inhibition of growth factors (Paubert-Braquet et al., 1996; Vacherot et al., 2000). Nevertheless, these effects are supported only by *in vitro* enzymatic studies, while the true *in vivo* mechanism has yet to be described (Geavlete, Multescu & Geavlete, 2011), and it is difficult to state which is the exact role of different active compounds (Lowe, 2001; Cabeza et al., 2003). It is the scope of this work to shed light on the possible mechanism of action of SRE on 5AR.

Although its full composition remains not completely known, SRE essentially consists of about 90% of free and esterified fatty acids, 6.8% of glycerides, 2.3% of unsaponified matter of which less than 0.3% consist of phytosterols (Habib & Wyllie, 2004; Buck, 2004) (see Table 1).

**Table 1:** SRE chemical composition.

Compound	%
Oleic acid	32
Lauric acid	29
Myristic acid	11
Palmitic acid	9
Linoleic acid	0.5
Methyl and ethyl esters	2.5
Long-chain esters	1.36
Glycerides	6.8
Unsaponified matter	2.27
*β*-sitosterol and daucosterolo	0.25
Stigmasterol	0.07
Campesterol	0.02

Both lipids and phytosterols are known to have significant biological properties and they are used as nutraceuticals and drugs for the prevention and treatment of several important diseases (Dalen & Devries, 2014; Mooradian & Haas, 2014; Morley, 2014). However, there is a lack in studies of single SRE component activity in the treatment of BPH, as the therapeutic effect is attributed to the whole phytocomplex.

Enzymatic *in vitro* studies demonstrated the ability of SRE and its components to inhibit 5AR. In 2008, Scaglione et al. reported that different compositions of the extract led to differences in 5AR inhibition potency. In a coculture model of epithelial and fibroblast cells, 10 µg/mL SRE showed an effective inhibition of both 5AR isoforms in epithelial cells and a selective inhibition of type II isozyme in fibroblast (Bayne et al., 1999). *β*-sitosterol alone has been shown to inhibit 5AR obtained from hamster prostate tissue with an IC_50_ of 2.7 µM (Cabeza et al., 2003). Abe et al. (2009) performed inhibition studies by using SRE and its components on type I and type II 5AR. As a result, SRE (40–300 µg/mL ) inhibited both the isoforms with an IC_50_ of 101 µg/mL. A similar result was found for most of the isolated fatty acid (10–300 µg/mL ), that showed an IC_50_ between 42 and 68 µg/mL. In particular, linoleic acid was found to be the most potent inhibitor. However, being oleic acid approximately 6-fold more abundant in SRE, it is expected to be 3–5-fold more active than linoleic acid. In 2002, following a similar experimental protocol, Raynaud et al. demonstrated that enzyme inhibition depends on the chain length and saturation state of the fatty acid. Effectively, compounds bearing saturated short chains were active toward both isoforms, while compounds with unsaturated chains were more potent and selective toward type I 5AR (Raynaud, Cousse & Martin, 2002). Contrary to Cabeza et al. (2003), they did not find any inhibition activity for SRE phytosterols.

Niederprüm, Schweikert & Zänker (1994) observed that linoleic acid is more potent than oleic acid and saturated fatty acids, and stated that 5AR inhibition depends on the chemical structure of fatty acids, with unsaturation being critical for the activity. They hypothesized that hydrophobic interactions between fatty acids and the enzyme are a mandatory condition for activity, and that unsaturated fatty acids could strongly interact with the enzyme, through the carbon–carbon double bond.

However, the possible mechanism of inhibition attributed to the fatty acids fraction is not well defined. Most of the studies speculate that fatty acids inhibit 5AR by altering the composition of the cell membrane to which the enzyme is functionally related (Abe et al., 2009), but there is a lack in knowledge about molecular interaction between SRE compounds and 5AR.

The attempt of this study is to evaluate the possibility that every single component of SRE may have an inhibitory effect on 5AR, by competitive interaction with testosterone for the binding site of the enzyme.

To test this hypothesis, we conducted computational tests, including molecular modeling and docking studies. It has been possible to dock all lipids and sterols of SRE in the active site of 5*β*-reductase (5BR) crystal, and thus speculate on each binding affinity among 5AR putative active site. In turn, the computed binding affinities allow us to make hypotheses on the overall effect of SRE in the treatment of BPH.

## Materials and Methods

X-ray crystal structures of 5AR are not available, and a BLAST search gave no significant sequence similarity. Functional similarities are known with 5BR (Yao et al., 2011), the structure of which is well known and present in the PDB database. Furthermore, 5AR and 5BR share the same substrate (testosterone) and inhibitor (finasteride). For this work the crystal structure of 5BR and its complex with NADP^+^ and testosterone (PDB: 3BUR (Di Costanzo et al., 2008)), and with NADP^+^ and finasteride (PDB: 3GIR (Drury et al., 2009)) were used. A homology model of 5AR obtained by Min-Rui and Jun-Qian, built using 5AR type 1 sequence as the target sequence and 3BUR as the template, is also available (Min-Rui & Jun-Qian, 2012) and was also used.

Chain A from 3BUR (326 aminoacids) was selected, and used as apo-protein through Pymol (PyMOL Molecular Graphics System, Version 1.3rl; Schrödinger, LLC, Portland, OR, USA): all non-catalytic waters and glycerols were removed and hydrogen atoms were added.

The structures of NADP^+^, testosterone and finasteride were saved from the pdb files, whereas the structures of SRE compounds of interest were obtained in two different ways: *β*-sitosterol, stigmasterol, campesterol and daucosterol were modelled on the basis of testosterone, whereas the structures of oleic, lauric, myristic, palmitic and linoleic acids were modelled from scratch and minimized using Avogadro (Hanwell et al., 2012) software. Structures were graphically displayed, modified and evaluated using Pymol (PyMOL Molecular Graphics System, Version 1.3rl; Schrödinger, LLC, Portland, OR, USA) and PyRosetta (Kaufmann et al., 2010).

To validate the docking protocol, self-docking of testosterone and finasteride in the relative x-ray structure (3BUR and 3G1R) was performed and RMS was calculated through Pymol. Re-docking of testosterone in 3BUR gave a RMS of 0.103 Å, whereas re-docking of finasteride in 3G1R gave an RMS of 0.124 Å, thus confirming the validity of the docking protocol.

As phytosterols structures were built by simply adding or removing substituents from testosterone and finasteride structures retrieved from 3BUR and 3G1R pdb files, the coordinates of the steroid nucleus were kept unvaried. Phytosterols were therefore put into the catalytic site manually, using the same coordinate of testosterone and finasteride from the crystal structures, and automatically by docking studies realized with AutoDock 4.2.5.1 and AutoDockTools 1.5.6 (Morris et al., 2010). Fatty acids were docked by means of AutoDock and AutoDockTools.

Complexes obtained from manual and AutoDock docking were submitted to optimization through PyRosetta 1.0.

**Figure 3:**
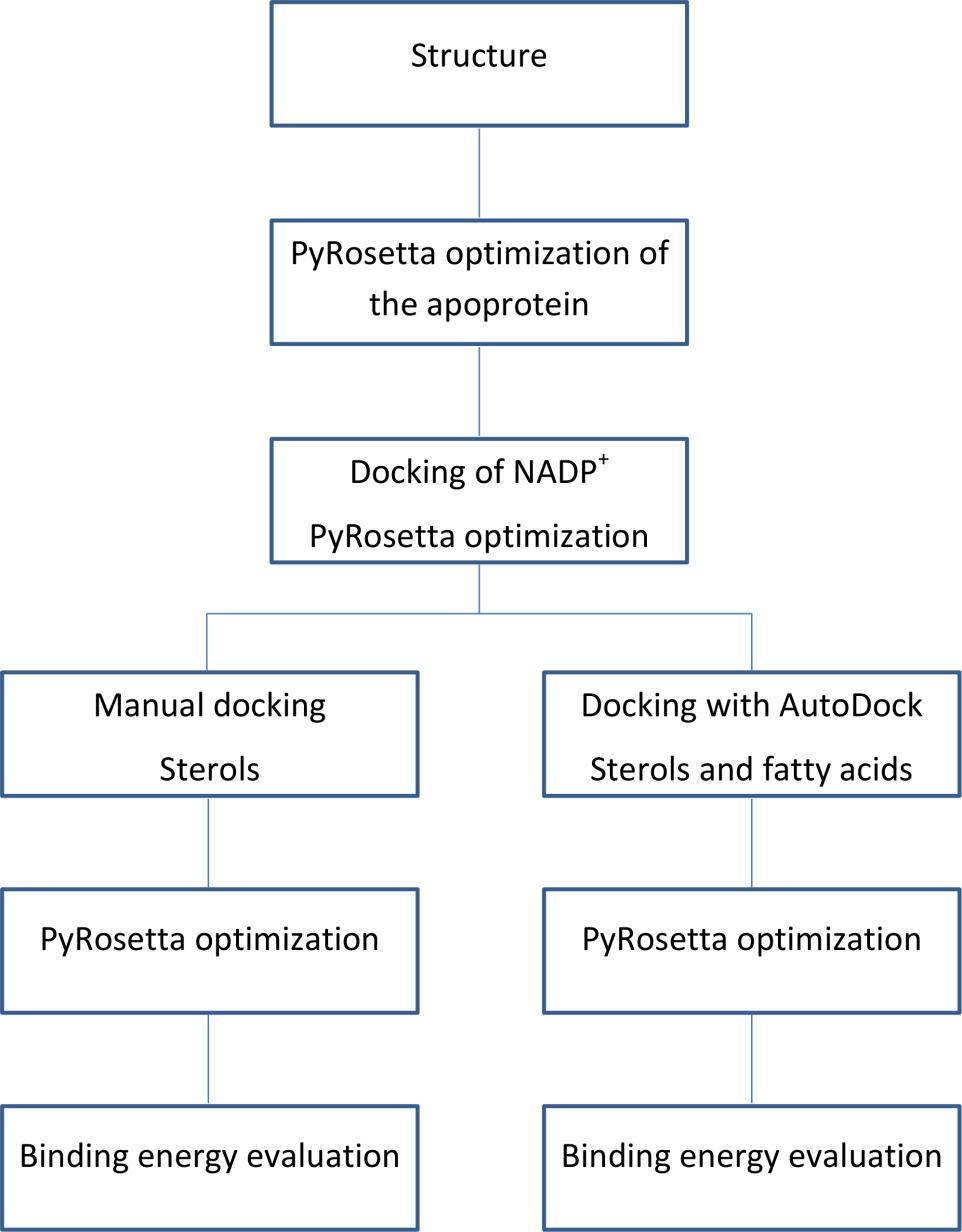
Calculations process flowchart.

The flowchart of the calculations is shown in Fig. 3.

To further validate our experimental protocol, a set of decoy ligands was generated using DUD-E (Mysinger et al., 2012). The top six decoy ligands generated by DUD-E were docked in both the productive and the unproductive position and submitted to PyRosetta optimization rounds. The binding energies of the top 6 decoys generated by DUD-E are reported in Table S5.

After PyRosetta based energy optimizations, polar and hydrophobic interactions between the ligand and the enzyme were visualized through LigPlot^+^ (Laskowski & Swindells, 2011).

All calculations were performed on personal computer and on the sunray cluster available at the department of chemistry, University of Copenhagen.

### PyRosetta algorithm

Crystal structures are only available for two of the tested ligands (testosterone and finasteride). In the attempt of making complexes generated after docking simulation, as well as computed binding energies, comparable to each other, we applied computational optimizations through PyRosetta software.

**Figure 4:**
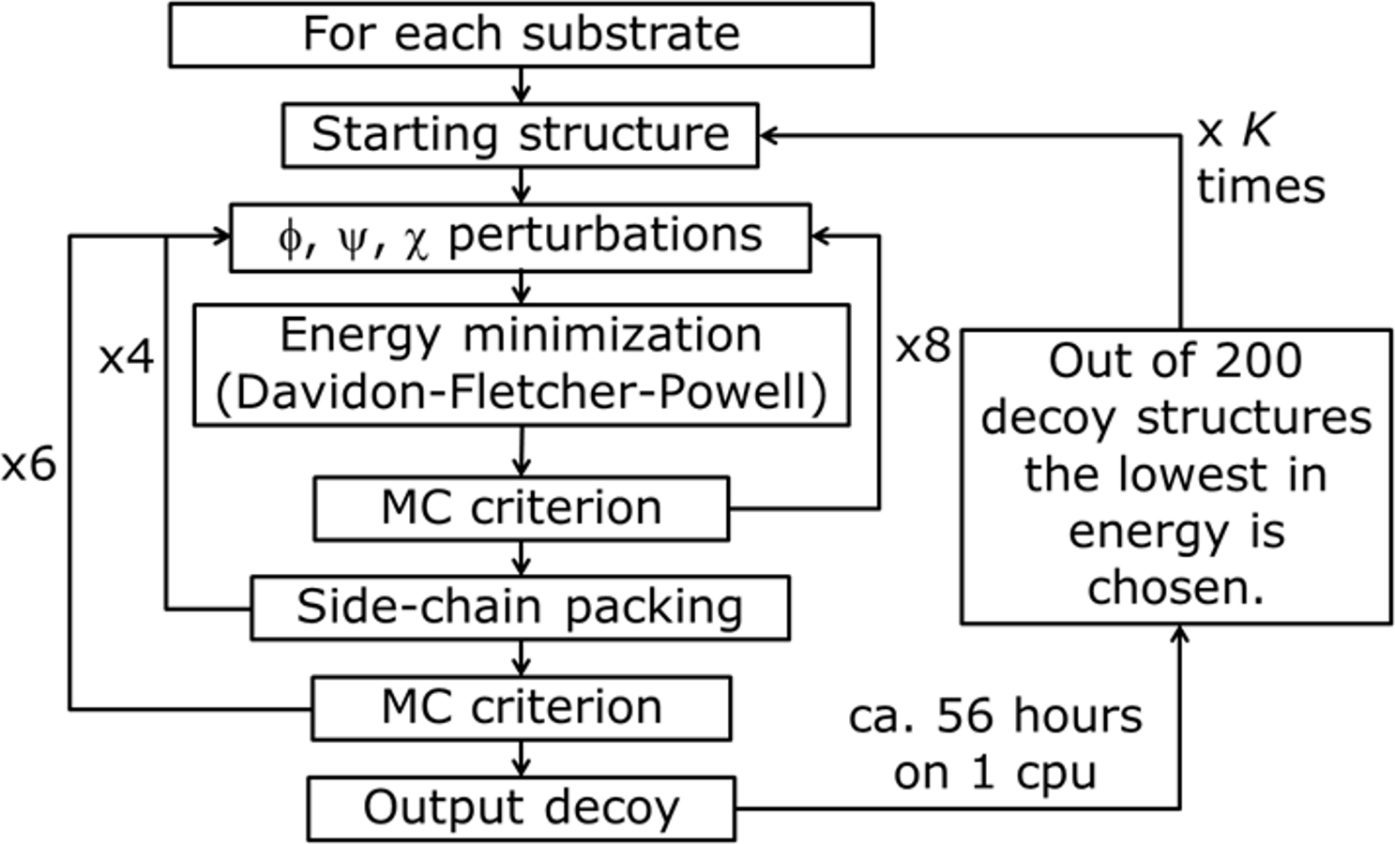
PyRosetta based optimization algorithm. *φ*, *y*, *χ* represent perturbation applied to both backbone and side chain dihedral angles. MC criterion stands for Monte Carlo based check of decoy structure.

The used algorithm, executed in PyRosetta 1.0 and described in Fig. 4, is based on the flexible peptide docking algorithm used by Chaudhury & Gray (2009), but, with respect to that, there are some differences: a larger number of cycles (8 × 4 × 6 = 192 compared to 8 × 12 = 96) and more “small” and “shear” moves for the perturbation of both side chain and backbone atoms are performed (Jensen et al., 2014). The side chain conformations are further optimized through a repacking algorithm (Kuhlman & Baker, 2000) and using the extended Dunbrack library (Dunbrack & Cohen, 1997; Wang, Schueler-Furman & Baker, 2005). The moves are applied to all substrates, including NADP^+^ and the catalytic water, plus a selected number of residues of the enzyme, with the following criterion: for the first cycle of optimization, all residues within a 4 Å distance from any atom of the NADP^+^ and the substrate, including all the residues reported as active by Di Costanzo et al. (2008), Di Costanzo et al. (2009) and Drury et al. (2009); for all the following cycles, only residues inside a 4 Å distance from any atom of the substrate. After the moves, an energy minimization step was executed, based on the Davidon–Fletcher–Powell method (Fletcher & Powell, 1963; Davidon, 1991). Each structure is then accepted or rejected based on a Monte Carlo criterion depending on the standard RosettaDock energy function (Dunbrack & Cohen, 1997; Lazaridis & Karplus, 1999; Kuhlman & Baker, 2000; Kortemme & Baker, 2002; Gray et al., 2003). Along the optimization a temperature gradient was applied, from an initial value of kT = 3.0 to 1.0. 200 new model of the given protein, defined by Chaudhury and colleagues as “decoy structures” (Chaudhury, Lyskov & Gray, 2010), were generated using each algorithm run. Once the 200 decoy structures were produced, the lowest in energy was chosen and used as a starting structure for another cycle of optimization. This process was repeated *K* times until convergence; at the end of the *K* cycles the resulting decoy was chosen as the PyRosetta optimized structure. Convergence was defined as when the computed energies of two subsequent cycles were the same.

**Figure 5:**
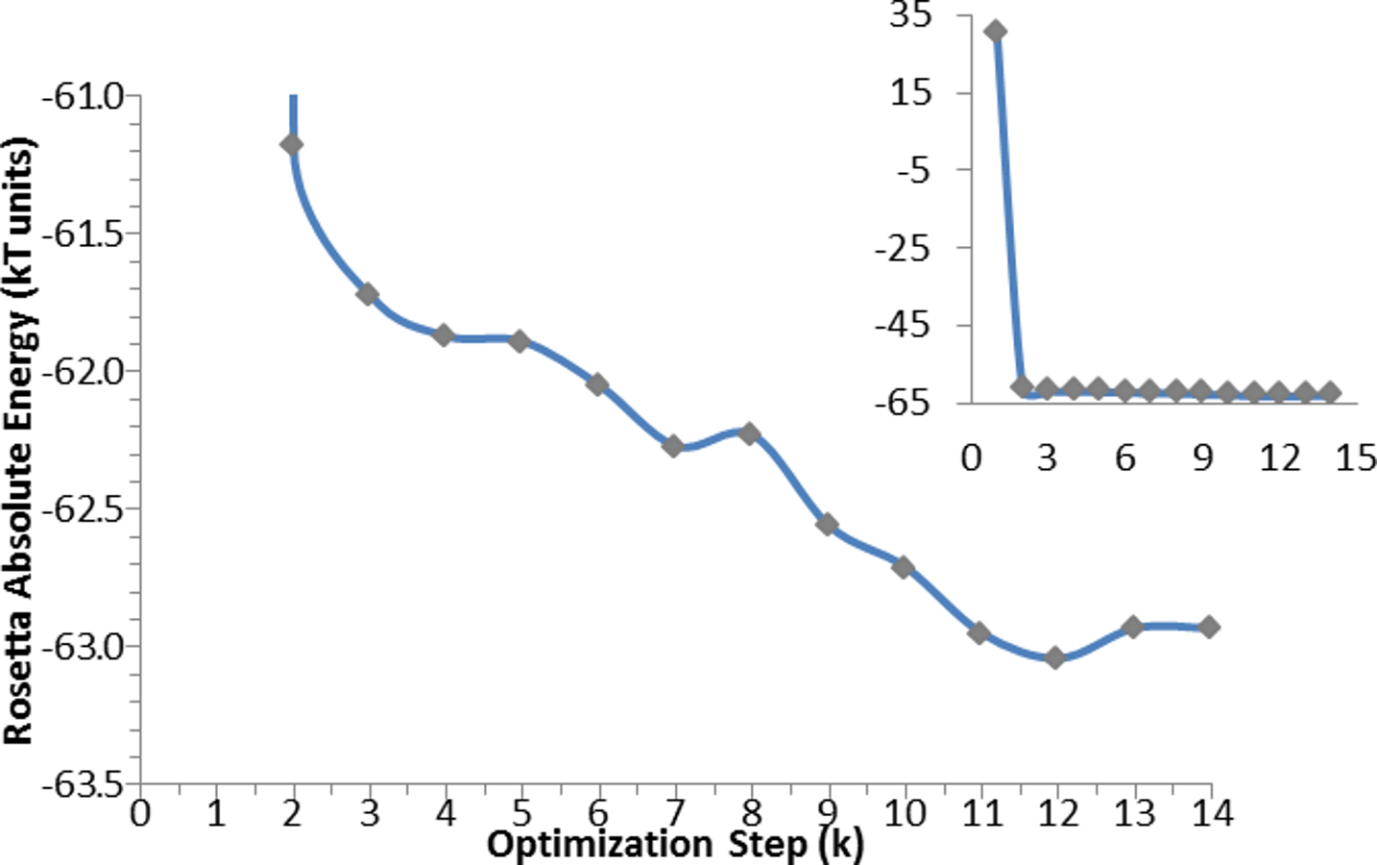
Optimization algorithm convergence. Energy convergence steps in the optimization algorithm for the apo-protein. Each point corresponds to the energy of the lowest in energy decoy out of the 200 produced during each of the *K* steps. The inset graph shows the total progress of the algorithm, whereas the central graph displays in details the optimization steps.

The same algorithm was used for the optimization of the apo-protein (see Fig. 5), and of the enzyme in complex with: catalytic water; catalytic water + NADP^+^; catalytic water + NAPD^+^ + substrate. For the substrate alone, only an energy calculation was performed.

### AutoDock calculations

The ligands were used as flexible molecule, and the torsion count together with the number of active torsion was automatically set; the macromolecules used were chain A from 3BUR and chain B from 3GIR, after PyRosetta optimization, containing NADP^+^ and the catalytic water.

The map type was defined based on the ligand, and the grid box was centered on the macromolecule and extended to the entire surface. For docking calculations, the genetic algorithm parameters were modified to get the maximum number of evals (25,000,000) and a Lamarckian genetic algorithm was performed. The results of the docking calculation were evaluated in AutoDockTools: the best pose, among the 10 different poses produced by AutoDock for each ligand, was selected on the basis of the graphical analysis of binding mode and interactions with residues in the binding sites.

### Evaluation of binding energies

As it was not possible to use a reasonable number of flexible residues in the enzyme for Autodock calculation, the binding energies obtained were not sufficiently reliable. Therefore, after every docking process, the obtained complexes were optimized through PyRosetta and binding energies were evaluated using PyRosetta computed energies.

The following equation was used to evaluate qualitative binding energies of the different ligands:

\begin{eqnarray*}{E}_{\mathrm{bind}}={E}_{\mathrm{complex}}-({E}_{\mathrm{NAP}+\mathrm{HOH}}+{E}_{\mathrm{lig}}) \end{eqnarray*}

where: *E*_bind_ is the binding energy; *E*_complex_ is the energy of the enzyme in complex with NADP^+^, catalytic water molecule and the ligand; *E*_NAP+HOH_ is the energy of the enzyme in complex with NADP^+^ andthe catalytic water molecule and *E*_lig_ stands for the energy of the ligand alone.

In computing and comparing these qualitative binding energies, we assume that every substrate has the same probability to reach the enzyme active site, and that overall entropic effects are nearly the same for every considered target molecule.

## Results and Discussion

An *in silico* structure-based approach has been applied in the attempt to dissect SRE activity toward 5AR and to identify single components able to bind to the enzyme. In particular, phytosterols, which are structurally similar to testosterone, and fatty acids, representing the major components of SRE, have been docked into the enzyme active site to check for their putative ability to block 5AR activity. However, due to the lack of any three-dimensional structure of this enzyme, only two strategies were applicable: to use homology modeling structures of 5AR or the available three-dimensional structure of a functionally similar protein.

**Figure 6:**
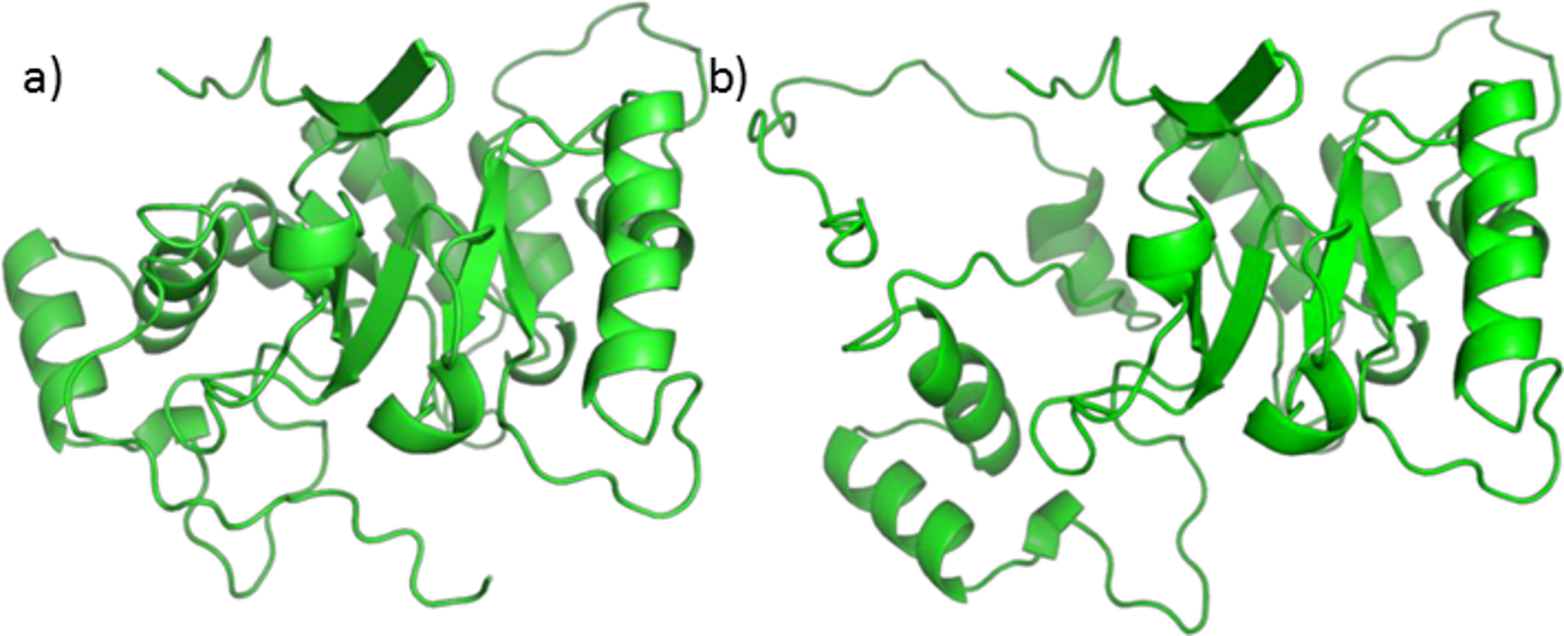
5AR model before (A) and after (B) optimization. The loss of structural features is evident.

A homology model, using 5AR type 1 sequence as target and 5BR crystal as the template already exists (Min-Rui & Jun-Qian, 2012), but its refinement does not seem to be trustworthy. Attempts to optimize the model alone, the binary complex with NADP^+^, or the ternary complex with NADP^+^ and one of testosterone, finasteride or SRE components through PyRosetta gave no reliable results. The structure of the model changed significantly upon optimization, and the protein secondary elements (such as alpha helices and beta sheets) suffered from structural limitations (see Fig. 6).

Moreover, the total energies were extremely high, reaching more than 4,000 kcal/mol. For these reasons our protocol was not applicable to the homology model and we decided to use a similar protein for this work. Nevertheless, we tried to perform Autodock calculations for NADP^+^ on the 5AR homology model; the obtained complex was then used to dock testosterone, finasteride and each of the SRE compounds of interest. Despite the doubts generated by the quality of the homology model, it is interesting that both sterols and fatty acids assumed similar positions and that AutoDock energies evaluation shows comparable affinity for every ligands Table S1.

5BR is, as 5AR, a member of the oxidoreductase family, using alkanes as electron donors and NADP^+^ as the acceptors (Enzyme Commission code: EC. 1.3.1). Although there is poor protein sequence similarity (approximately 10%), both 5AR and 5BR are NADP(H)-dependent and share a conserved nicotinamide-cofactor-binding pocket (Jez et al., 1997), same substrate (testosterone) and same inhibitor (finasteride), thus suggesting some similarities in their substrate binding domains (Langlois et al., 2010) and function (Min-Rui & Jun-Qian, 2012). Furthermore, the sequence alignment shows that 5BR binding site residues W89 and W230 are identical in both 5AR type 1 and type 2 and that the active residue Y58 is also conservatively substituted by a phenylalanine in the type 1 isozyme.

**Figure 7:**
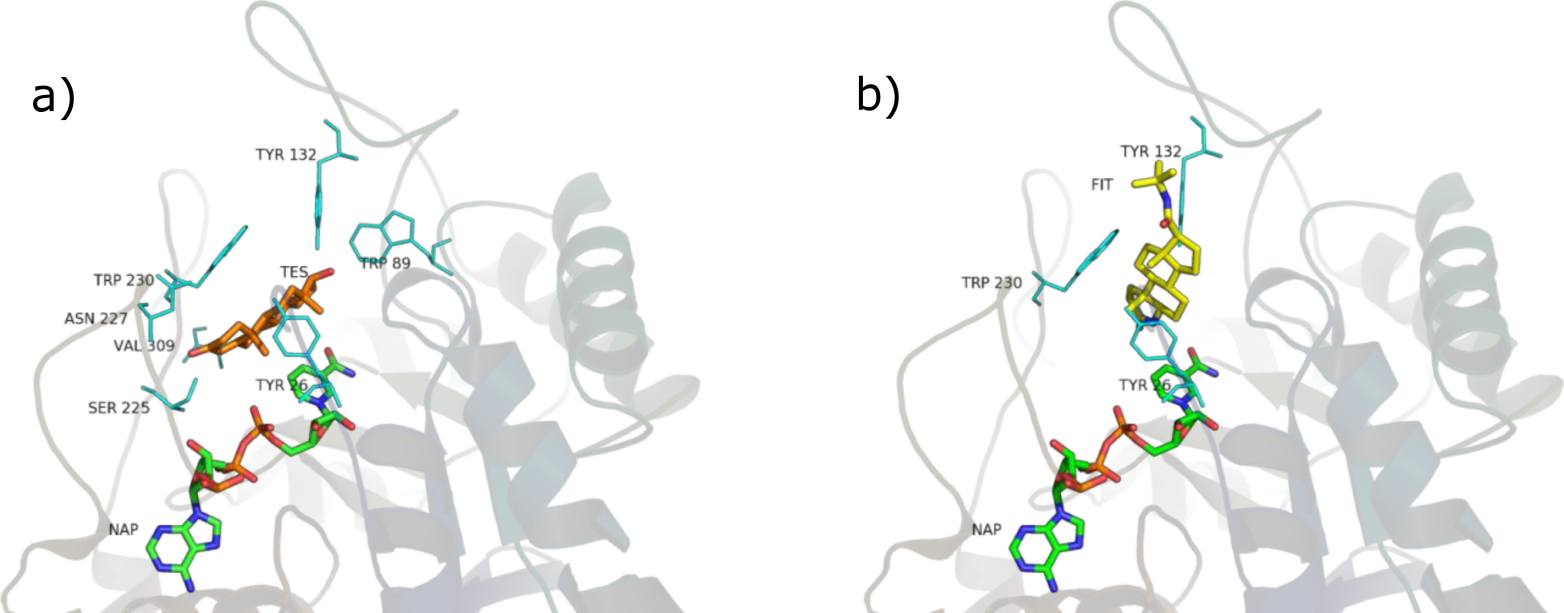
Testosterone in the unproductive position (A) and finasteride in the productive position (B).

Analysis of the available crystal structures of 5BR and its ligands showed that testosterone and finasteride can assume two different orientations: one is judged as productive (capable of carrying out the double bond reduction), perpendicular with respect to NADPH, and the other as unproductive (unable of carrying out the double bond reduction), parallel to NADPH. Both positions are shown in Fig. 7. Finasteride has been crystalized in the productive position (Drury et al., 2009) whereas it was not possible to obtain the crystal structure of the productive position assumed by testosterone Di Costanzo et al. (2008) assumed that testosterone could occupy more than one position and that at the high steroid concentration used in the crystallization trials, the unproductive binding mode is favored. In our opinion, this fact has at least two consequences: (i) when binding in the productive position, testosterone quickly reacts and is subsequently released, making it very difficult to obtain a crystal structure of an active enzyme; (ii) we suppose that the unproductive position could work as a regulatory element of the enzyme activity. If testosterone could not bind also to this position the enzyme could possibly be misregulated.

### Protocol validation

Testosterone and finasteride were docked in both productive and unproductive positions and the binding energies were computed through the PyRosetta scoring function (Table 2).

**Table 2:** Testosterone, finasteride, SRE phytosterols and SRE fatty acids binding energies (kcal/mol) obtained with PyRosetta, using 5BR as a target protein.

Substrates	Unproductive position	Productive position
Testosterone	−7	−11
Finasteride	−6	−27
*β*-sitosterol	−2	−28
Stigmasterol	0.1	−29
Campesterol	0.5	−8
Daucosterol	−34	−30
Oleic acid	−12	−34
Lauric acid	−3	Not binding
Myristic acid	−8	−20
Palmitic acid	−12	−13
Linoleic acid	−7	−28

According to PyRosetta-computed binding energies, testosterone and finasteride have the same affinity for the unproductive position, whereas finasteride is a better candidate for binding the productive position, thus confirming the effectiveness of finasteride as a 5AR-inhibitor already known from clinical experience.

PyRosetta effectiveness in predicting the correct binding energies was validated by docking 6 automatically generated ligands in the productive and unproductive positions. PyRosetta algorithm efficiently addressed the DUD-E generated inactive ligands, for which it computed worse binding energies Table S5 than those of testosterone, finasteride and SRE components.

**Figure 8:**
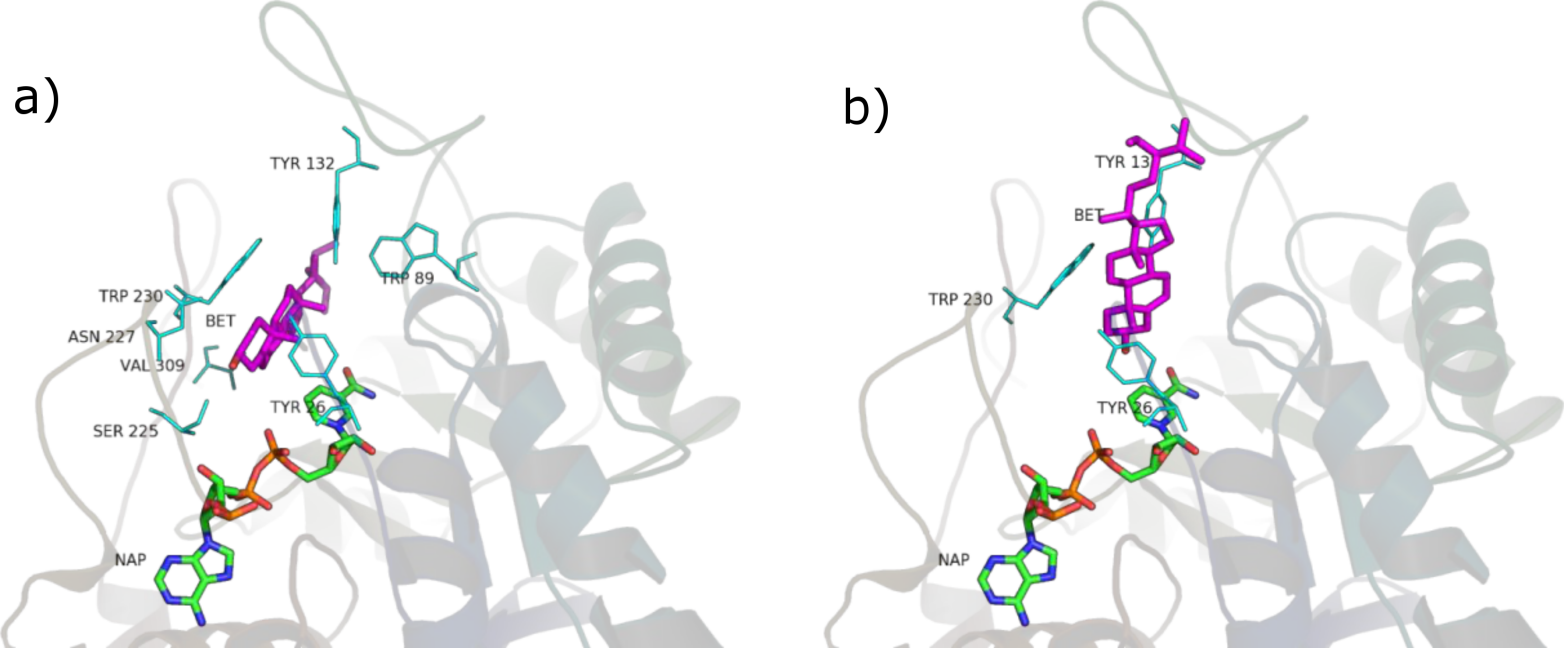
*β*-sitosterol in the unproductive (A) and productive (B) position.

### Phytosterols

SRE phytosterols’ (Table 1) structures are very similar to that of the endogenous substrate of the enzyme (see Fig. 2), so it has been possible to perform manual and AutoDock docking simulations. As expected, *β*-sitosterol, stigmasterol and campesterol bind the active site of 5BR in the two possible orientations (Fig. 8 and Fig. S1).

PyRosetta-computed binding energies after AutoDock docking resulted higher, although comparable, than those calculated after manual docking Table S4; for this reason, in this work SRE phytosterols–5BR complexes resulting from manual docking were chosen, when comparing with other complexes.

Considering PyRosetta binding energies (Table 2), *β*-sitosterol, stigmasterol and daucosterol resulted to be the best candidates to compete with testosterone for the active site of the enzyme, giving a binding energy comparable to that of finasteride. Campesterol is comparable to testosterone.

**Figure 9:**
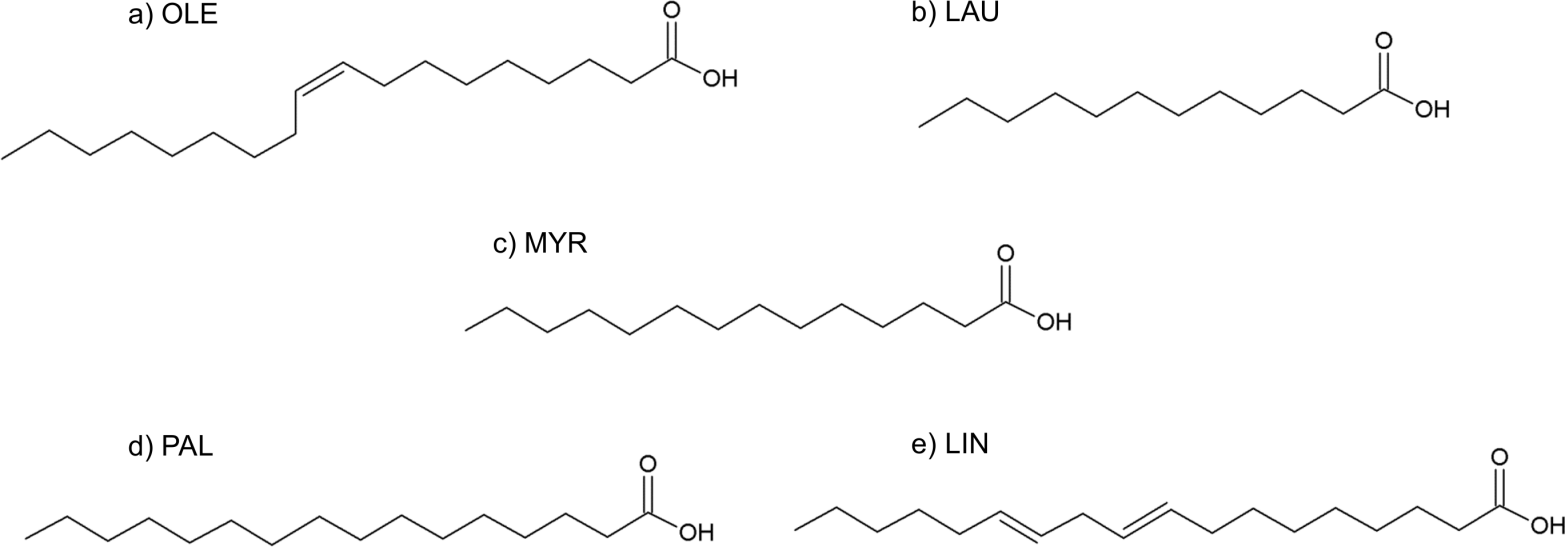
Formulas of (A) oleic acid, (B) lauric acid, (C) myristic acid, (D) palmitic acid, (E) linoleic acid.

### Fatty acids

**Figure 10:**
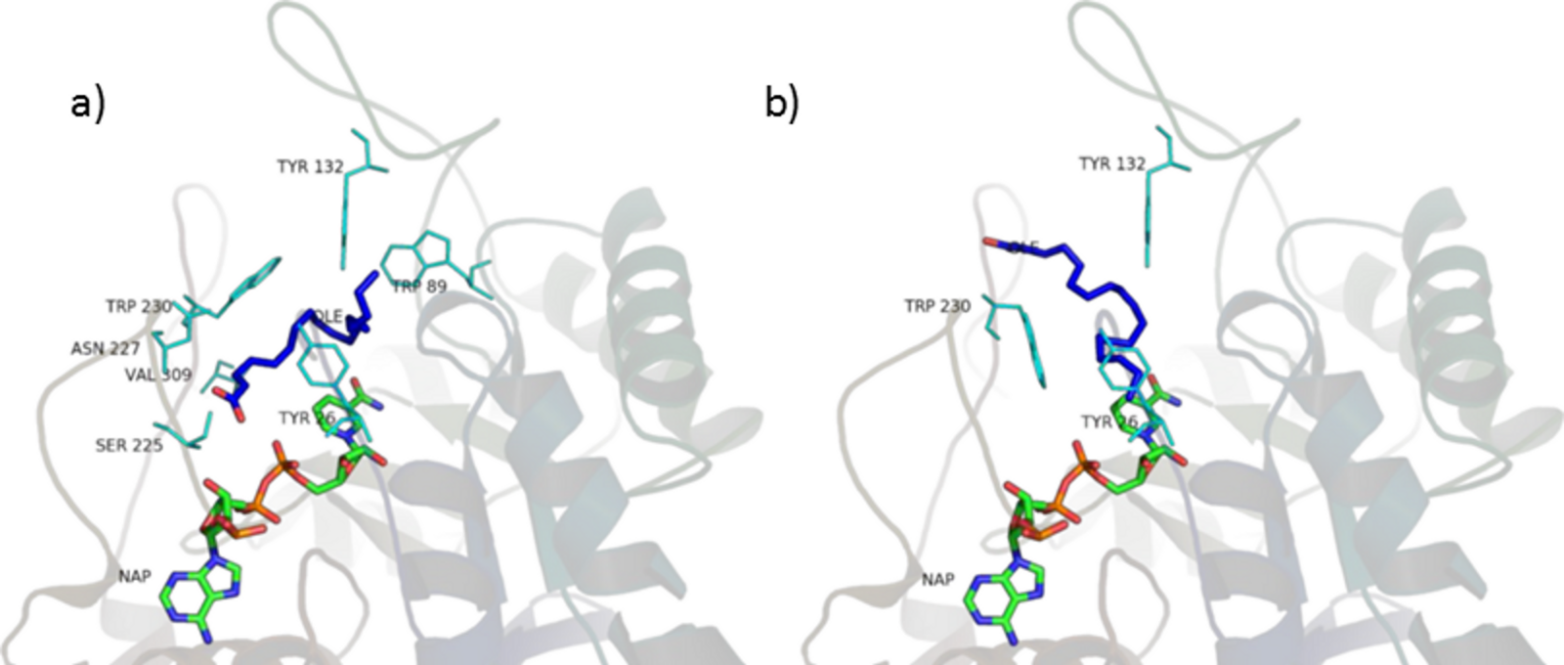
Oleic acid in the unproductive (A) and productive (B) position.

The fatty acids contained in SRE (see Table 1 and Fig. 9) were docked in the active site of the enzyme by means of AutoDock: although completely different in chemistry and structure, they were found to be able to dock in both productive and unproductive positions (Fig. 10 and Fig. S2) and most of the interactions (see later) were conserved, thus suggesting a possible competitive mechanism of inhibition.

According to PyRosetta binding energies (reported in Table 2), oleic and linoleic acid seem to be the best competitor (comparable to finasteride), whereas myristic and palmitic are similar to testosterone. Lauric seems to be useless, as it cannot bind the productive position.

### Residues interactions

The main interactions of the obtained complexes, resulted from LigPlot+ evaluations, are shown in Tables S3 and S4. In the unproductive position, interactions with Y26, W89, Y132, S225, N227, W230, V309 are shared by almost every ligand. In the productive position, interactions with Y26, Y132 and W230 are shared by every ligand binding in this position.

Considering these results, it could be said that interactions with Y26, Y132 and W230, which are cavity residues, are important for ligand binding to the active site and this has to be taken into consideration when studying a pharmacophore model for the inhibition of this enzyme. Furthermore, W230 is a conserved residue in both the isozymes of 5AR (see sequence alignment in Figs. S3 and S4), suggesting an important role for the binding of ligands to the active site of 5AR too.

Although the tested ligands are mainly hydrophobic, some of them have polar and hydrophilic substituents in their structures. This fact seems to be important for the correct alignment in the binding pocket and for the binding energy scoring. In particular, in the unproductive position, testosterone and finasteride established a hydrogen bond between the oxygen atom in 3 and residue S255. This kind of polar interaction is conserved by the non-glycosilated phytosterols (*β*-sitosterol, stigmasterol, campesterol). The carboxyl group present in the tested fatty acids also established hydrogen bond with S225. Furthermore, lauric, linoleic and myristic acid made polar interactions with R226, and oleic, lauric and linoleic acid with N227. Palmitic acid made no hydrophilic interactions.

In the productive position, instead, testosterone and finasteride made no polar contacts with residues in the active site, and this is shared by the tested phytosterols. Oleic and linoleic acid, on the contrary, established hydrogen bonds between the carboxyl group and residues E310 and L311. This could be an explanation for their better binding energies with respect to the other ligands.

### Bioavailability

The recommended dosage of *Serenoa repens* extract is 320–640 mg/day; phytosterols concentration reaching the prostate after oral treatment at therapeutic dosage of SRE is expected to be comparable to that of finasteride (Duchateau et al., 2012; Merck & Co., 2013).

Little information is available in literature about dietary fatty acids distribution in prostate; nevertheless, they are expected to reach the prostate in high concentration, on the basis of some studies carried on SRE and similar extracts pharmacokinetics (De Bernardi di Valserra, Tripodi & Contos, 1994; Bernard, Cousse & Chevalier, 1997; Yohani, Men & Rosa, 2006).

In this work, daucosterol was included in the set of substrates for docking calculation, even if it is a glucoside, hence it cannot reach the prostate as such but as *α*- and *β*-sitosterol. Daucosterol binding position differs from that assumed by the non-glucoside phytosterols, due to the presence of the glucose portion. Nevertheless, its binding energy resulted to be comparable to that of finasteride, *β*-sitosterol and stigmasterol, suggesting that it could be used as a 5AR inhibitor by means of currently known nanodevices specific drug-delivery systems.

Making the hypothesis that 5AR and 5BR share mechanisms of regulation as they share many features of the binding pocket and function, given the expected concentration of SRE phytosterols reaching the prostate after oral delivery (Duchateau et al., 2012; Merck & Co., 2013), and the computed qualitative binding energies reported in Table 2, we can reasonably assume that SRE phytosterols have a similar inhibition action on 5AR to that of finasteride. Published experimental tests to confirm this hypothesis are controversial: Cabeza et al. (2003) observed the inhibitory effect of *β*-sitosterol alone on 5AR, and this is in agreement with our findings. Nevertheless, Raynaud and colleagues observed no inhibitory effect for the unsaponified matter (i.e., containing phytosterols) of SRE (Raynaud, Cousse & Martin, 2002).

On the basis of our computational results, long chain and unsaturated fatty acids, like oleic and linoleic acid, are the best candidates from SRE to act as competitive inhibitors of 5AR with respect to saturated and short/middle chain fatty acids. These results are in agreement with those reported by previously conducted *in vitro* experiments (Niederprüm, Schweikert & Zänker, 1994; Raynaud, Cousse & Martin, 2002; Abe et al., 2009).

However, fatty acids are a very common food intake and testosterone is expected to compete with them regularly, being the amount of fatty acids taken up by diet much greater than that taken by therapeutic dosage of SRE.

SRE may be clinically effective because of the free form of the fatty acids. Food intake is predominantly composed of esterified fatty acids: free fatty acids may be assimilated by the digestive system in a higher concentration (Niederprüm, Schweikert & Zänker, 1994). This hypothesis is confirmed by the fact that another phytotherapic treatment used in the tradition of many countries to relieve LUTS is the oil obtained from *Cucurbita pepo* L. seeds (Allkanjari & Vitalone, 2015; European Medicines Agency, 2010), which contains a high amount of free fatty acids (Badr, Shaaban & Elkholy, 2011).

However, the reason behind lower incidence of secondary effects from SRE than from finasteride treatment remain to be understood, as they are possibly related to further mechanisms of action of SRE.

## Conclusions

*Serenoa repens* has been used to treat the symptoms related to BPH for long time, thanks to its large set of mechanisms of action and to its handed down information on effectiveness.

Within the variety of mechanisms of action, the inhibition of 5AR seems to have an important role and has been demonstrated by many studies; nevertheless, a competitive mechanism of inhibition has never been speculated, as to SRE only an action toward cell membrane to which 5AR is functionally related has been attributed.

In this work, an *in silico* approach has been applied to study the 5AR molecular mechanism of inhibition induced by SRE in depth. The tridimensional structure of 5AR has not been described to date, but many of the binding site features are conserved in the available 5BR; until a better-refined structure of 5AR will be available, 5BR is the most reliable asset to use for these kind of studies.

SRE phytosterols and free fatty acids have been demonstrated to be good inhibitors for 5AR in enzymatic tests (Niederprüm, Schweikert & Zänker, 1994; Raynaud, Cousse & Martin, 2002; Cabeza et al., 2003; Abe et al., 2009) and are supposed to have a good prostatic bioavailability: this work proposes a competitive mechanism of inhibition, considering their affinity for the active site of 5BR.

Computed qualitative binding energies of testosterone, finasteride and SRE components show that: (i) the phytosterols part can act as inhibitor just like finasteride (Table 2), also considering the expected concentrations in the prostate tissues; (ii) the fatty acid fraction can also act as inhibitor (Table 2), as previously demonstrated by the *in vitro* experiments. How they can arrive to the prostate and what differentiates them from fatty acids in food intake is still to be examined.

Future development of a better-refined structure of 5AR is an exciting prospect for better understanding molecular mechanism of action of synthetic 5AR inhibitors and phytotherapics like SRE, and would allow repeating this study comparing differences and similarities with 5BR.

Several pharmacophoric models for 5AR inhibitors have been already reported in the literature. In particular, Chen et al. (2001) described a hypothetical pharmacophore for 5AR inhibitor constituted by two hydrogen bond acceptors (HBA) and three hydrophobic features. In 2003, Faragalla et al. developed several pharmacophores for human and rat type I and type II 5AR, and compared them to those obtained earlier by Chen. In detail, they described two different hypotheses for human type II 5AR inhibitors: hIIA consisted of one HBA, a negative ionizable group and two ring aromatic features, and was the most similar to that obtained from Chen; hIIB was however considered more reliable by the authors and consisted of one HBA, one hydrophobic and two ring aromatic features.

An analysis of our results showed that the hydrophobic features are maintained by the steroid core of phytosterols and by the long aliphatic chain of fatty acids. One of the HBA reported by Chen corresponds to the substituent in position 3 of the steroid structure. Moreover, each of the steroid ligands (testosterone, finasteride and phytosterols), docked in the unproductive position, made a hydrogen bond involving its substituent in position 3 and the portion of the binding site comprising S225, R226 and N227. This hydrogen bond is also conserved by the carboxyl group of the tested fatty acids, apart from palmitic acid.

In addition, two recent 3D-QSAR studies indicated that C17 substituents are responsible for the hydrophobic interactions of steroid inhibitors with the active site of 5AR, thus suggesting that bulky hydrophobic substituent at C17 are necessary for inhibitory activity (Kumar et al., 2013; Thareja, Rajpoot & Verma, 2015). In agreement with these findings, our results confirmed that many hydrophobic interactions are established between phytosterols C17 substituent and 5BR active site, both in the unproductive and productive positions. Long chain fatty acids, such as oleic and linoleic acid, share similar hydrophobic interactions, and this could explain their better *in vitro* inhibitory activity and calculated binding energies. More studies are required to confirm and further investigate the results here presented; comparisons between the effects of free and esterified fatty acids would be particularly useful to explain the difference in the effectiveness of SRE with respect to common food intake. Also, as one limit of this study is due to the lack of consideration of enthropic effects, we hope that more rigorous calculations of free binding energies will be conducted in the future. Nevertheless, the described method will be useful to test the mechanism of action of other phytotherapic treatments traditionally used in the management of BPH, such as *Prunus africana* (Hook. f.) Kalkman, *Urtica dioica* L., *Cucurbita pepo* L. and many others.

Furthermore, other aspects to be investigated about SRE mechanism of action (e.g., Aromatase inhibition) will benefit from *in silico* studies.

##  Supplemental Information

10.7717/peerj.2698/supp-1 Table S1 Autodock binding energy evaluation of 5AR homology model in complex with different substrates (kcal/mol)

10.7717/peerj.2698/supp-2 Table S2 PyRosetta computed absolute energies and computed binding energies (kcal/mol)

10.7717/peerj.2698/supp-3 Table S3 Unproductive position interactions

10.7717/peerj.2698/supp-4 Table S4 Productive position interactions

10.7717/peerj.2698/supp-5 Table S5 DUD-E generated decoys binding energies (kcal/mol) obtained by PyRosetta, using 5BR as a target protein

10.7717/peerj.2698/supp-6 Figure S1 Stigmasterol (a, b), campesterol (c, d) and daucosterol (e, f) in the unproductive (left) and productive (right) position

10.7717/peerj.2698/supp-7 Figure S2 Lauric acid (A, B), miristic acid (C, D), palmitic acid (E, F) and linoleic acid (H, I) in the unproductive (left) and productive (right) position

10.7717/peerj.2698/supp-8 Figure S3 5AR type 1 and 5BR sequence alignment Binding site residues are sharpened in light green; active site residues are sharpened in olive green.

10.7717/peerj.2698/supp-9 Figure S4 5AR type 2 and 5BR sequence alignment Binding site residues are sharpened in light green; active site residues are sharpened in olive green.

10.7717/peerj.2698/supp-10 Figure S5 Formulas of the top 6 decoys generated by DUD-E

## Additional Information and Declarations

### Data Availability

The following information was supplied regarding data availability: The research in this article did not generate any raw data.
