# Supplementary material for: Hypothesis on Serenoa repens (Bartram) small extract inhibition of prostatic 5α-reductase through an in silico approach on 5β-reductase x-ray structure
Source: PeerJ. 2016 Nov 22;4:e2698. doi: 10.7717/peerj.2698 (PMC5126621; doi:10.7717/peerj.2698)
Supplement: Table S1 [file peerj-04-2698-s001.pdf]

Supporting Table S1. Autodock binding energy evaluation of 5AR homology model in complex with different substrates (kcal/mol)

SUBSTRATES	ENERGY
$NADP^+$	-9
$NADP^+$ + testosterone	-8
$NADP^+$ + finasteride	-9
$NADP^+$ + beta-sitosterol	-11
$NADP^+$ + stigmasterol	-9
$NADP^+$ + campesterol	-10
*$NADP^+$ + daucosterol**	-10
$NADP^+$ + oleic acid	-8
$NADP^+$ + lauric acid	-8
$NADP^+$ + linoleic acid	-9
$NADP^+$ + palmitic acid	-8

*Bound in different site
