# Supplementary material for: Hypothesis on Serenoa repens (Bartram) small extract inhibition of prostatic 5α-reductase through an in silico approach on 5β-reductase x-ray structure
Source: PeerJ. 2016 Nov 22;4:e2698. doi: 10.7717/peerj.2698 (PMC5126621; doi:10.7717/peerj.2698)
Supplement: Table S2 [file peerj-04-2698-s002.pdf]

Supporting Table S2. PyRosetta computed absolute energies and computed binding energies (kcal/mol)

SUBSTRATES	UNPRODUCTIVE		PRODUCTIVE	
	Absolute	Binding	Absolute	Binding
**Testosterone**	-43	-7	-47	-11
**Finasteride**	-41	-6	-63	-27
**β-sitosterol AutoDock**	-30	-6	-51	-15
**Stigmasterol AutoDock**	-44	-7	-49	-12
**Campesterol AutoDock**	-41	-4	-50	-13
**Daucosterol AutoDock**	-58	-22	-44	-7
**β-sitosterol Manual**	-38	-2	-64	-28
**Stigmasterol Manual**	-36	0.1	-66	-29
**Campesterol Manual**	-36	0.5	-45	-8
**Daucosterol Manual**	-71	-34	-67	-30
**Oleic acid**	-47	-12	-69	-34
**Lauric acid**	-39	-3	Not binding	Not Binding
**Myristic acid**	-43	-8	-55	-20
**Palmitic acid**	-44	-12	-45	-13
**Linoleic acid**	-43	-7	-64	-28
