# Supplementary material for: Hypothesis on Serenoa repens (Bartram) small extract inhibition of prostatic 5α-reductase through an in silico approach on 5β-reductase x-ray structure
Source: PeerJ. 2016 Nov 22;4:e2698. doi: 10.7717/peerj.2698 (PMC5126621; doi:10.7717/peerj.2698)
Supplement: Table S3 [file peerj-04-2698-s003.pdf]

Supporting Table S3. Unproductive position interactions

SUBSTRATE / RESIDUE	TES	FIT	BET	STI	CAM	DAU	OLE	LAU	MYR	PAL	LIN
**Y26**	X	X	X	X	X	X	X		X	X	X
**E28**						X					
**P29**						X					
**I57**		X					X				X
**Y58**		X					X		X	X	X
**Q59**											
**W89**	X		X	X	X	X	X	X			X
**A90**					X						
**E120**					X	X	X	X	X	X	
**V121**			X	X	X			X	X		
**P122**			X								
**M123**			X	X							
**F125**			X	X	X						
**I131**	X	X			X		X				
**Y132**		X	X	X	X	X	X		X	X	X
**N170**			X								
**T224**		X			X		X	X	X	X	X
**S225**	X	X	X	X	X		X	X	X	X	X
**R226**		X			X			X	X	X	X
**N227**	X	X	X	X	X	X	X	X	X	X	X
**I229**						X					
**W230**	X	X	X	X	X	X	X	X	X	X	X
**V231**	X	X					X	X	X	X	X
**V309**	X	X	X	X	X		X	X		X	X
**L311**			X		X		X	X	X	X	X
**W314**			X	X	X				X	X	
