# Supplementary material for: Hypothesis on Serenoa repens (Bartram) small extract inhibition of prostatic 5α-reductase through an in silico approach on 5β-reductase x-ray structure
Source: PeerJ. 2016 Nov 22;4:e2698. doi: 10.7717/peerj.2698 (PMC5126621; doi:10.7717/peerj.2698)
Supplement: Table S4 [file peerj-04-2698-s004.pdf]

Supporting Table S4. Productive position interactions

SUBSTRATE / RESIDUE	TES	FIT	BET	STI	CAM	DAU	OLE	LAU	MYR	PAL	LIN
**Y26**	X	X	X	X	X	X	X		X	X	X
**E28**			X	X	X					X	
**P29**	X	X	X	X	X						
**K30**					X						
**I57**	X	X	X	X	X				X		X
**Y58**	X	X			X		X		X		X
**Q59**		X									
**W89**						X	X				
**V121**						X					
**I131**				X							
**Y132**	X	X	X	X	X	X	X		X	X	X
**N170**						X					
**Y219**						X					
**S225**										X	
**N227**										X	
**I229**							X				X
**W230**	X	X	X	X	X	X	X		X	X	X
**V309**						X			X		
**L311**						X	X		X		X
**L312**							X				X
**M313**							X				X
**W314**							X				X
