# Supplementary material for: Hypothesis on Serenoa repens (Bartram) small extract inhibition of prostatic 5α-reductase through an in silico approach on 5β-reductase x-ray structure
Source: PeerJ. 2016 Nov 22;4:e2698. doi: 10.7717/peerj.2698 (PMC5126621; doi:10.7717/peerj.2698)
Supplement: Table S5 [file peerj-04-2698-s005.pdf]

Supporting Table S5. DUD-E generated decoys binding energies (kcal/mol) obtained by PyRosetta, using 5BR as a target protein

SUBSTRATES	UNPRODUCTIVE POSITION	PRODUCTIVE POSITION
**DUD-E 1**	18	10
**DUD-E 2**	Not binding	-2
**DUD-E 3**	9	2
**DUD-E 4**	10	-7
**DUD-E 5**	15	0.1
**DUD-E 6**	10	-10
