# Supplementary material for: Hypothesis on Serenoa repens (Bartram) small extract inhibition of prostatic 5α-reductase through an in silico approach on 5β-reductase x-ray structure
Source: PeerJ. 2016 Nov 22;4:e2698. doi: 10.7717/peerj.2698 (PMC5126621; doi:10.7717/peerj.2698)
Supplement: Figure S1 [file peerj-04-2698-s006.pdf]

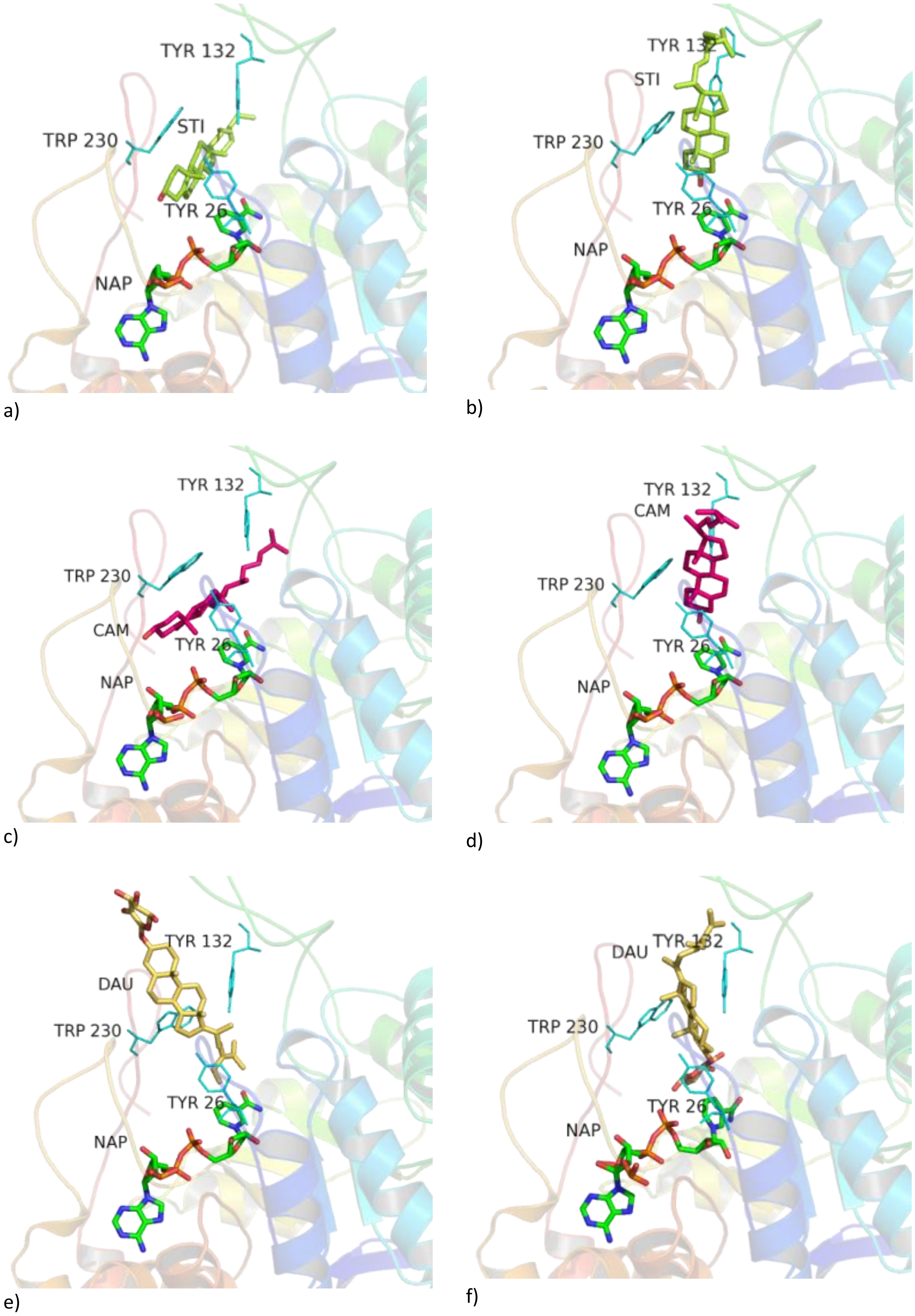

Supporting Figure S1. Stigmasterol (a,b), campesterol (c,d) and daucosterol (e,f) in the unproductive (left) and productive (right) position.

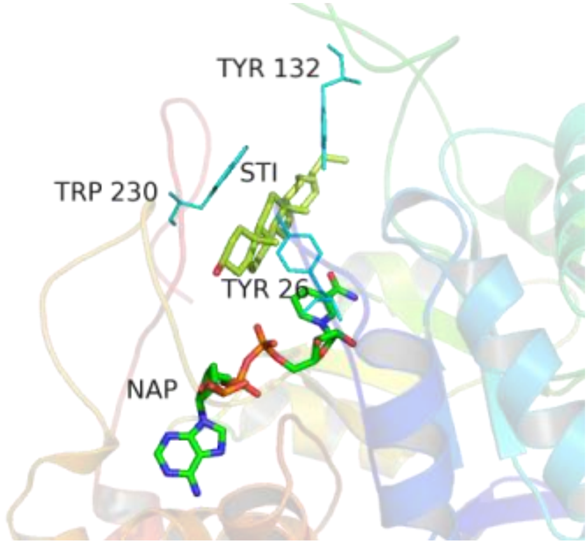

a)

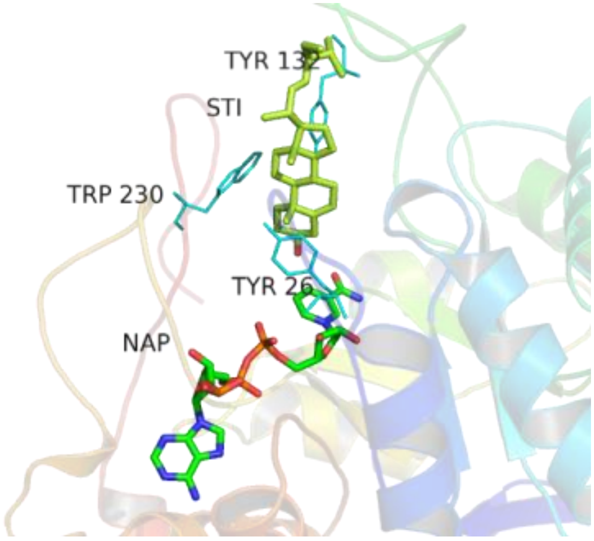

b)

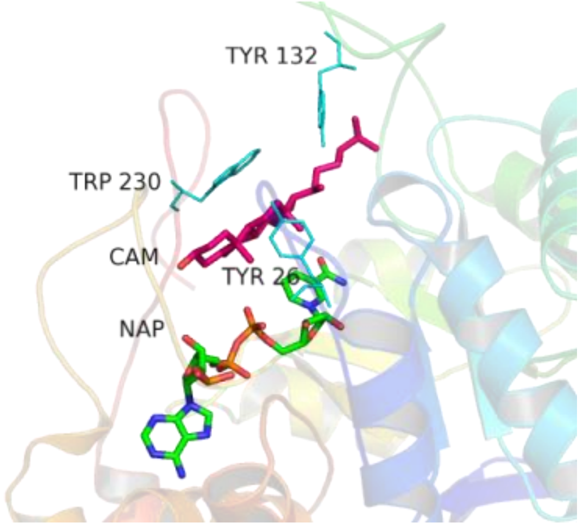

c)

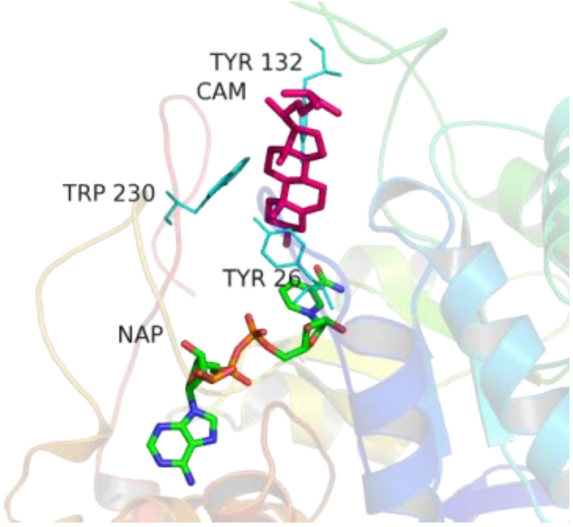

d)

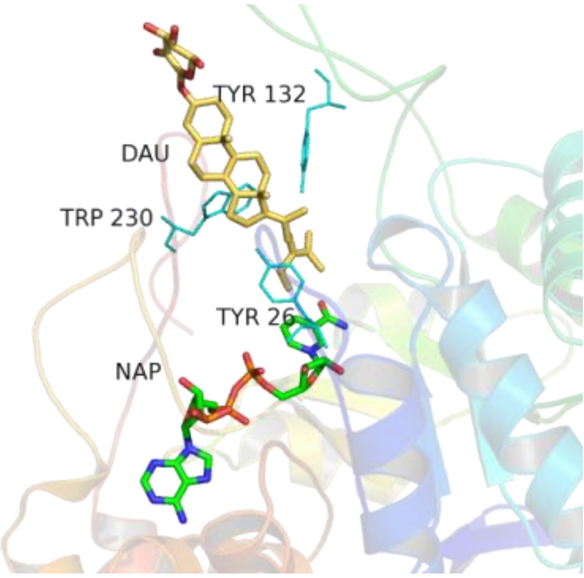

e)

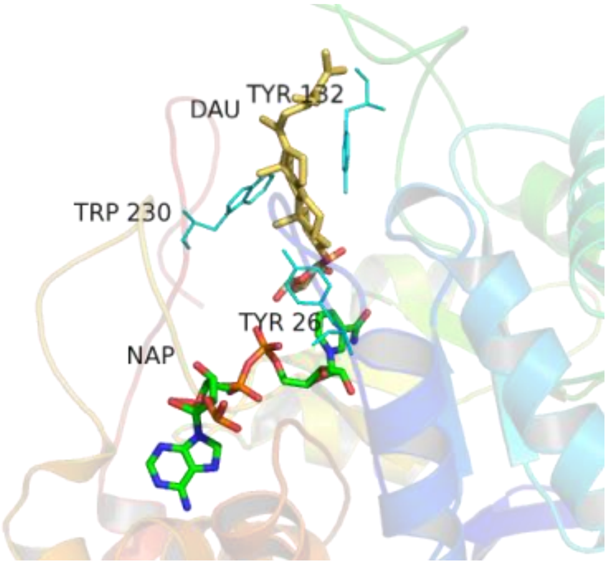

f)

Supporting Figure S1. Stigmasterol (a,b), campesterol (c,d) and daucosterol (e,f) in the unproductive (left) and productive (right) position.
