# Supplementary material for: Hypothesis on Serenoa repens (Bartram) small extract inhibition of prostatic 5α-reductase through an in silico approach on 5β-reductase x-ray structure
Source: PeerJ. 2016 Nov 22;4:e2698. doi: 10.7717/peerj.2698 (PMC5126621; doi:10.7717/peerj.2698)
Supplement: Figure S2 [file peerj-04-2698-s007.pdf]

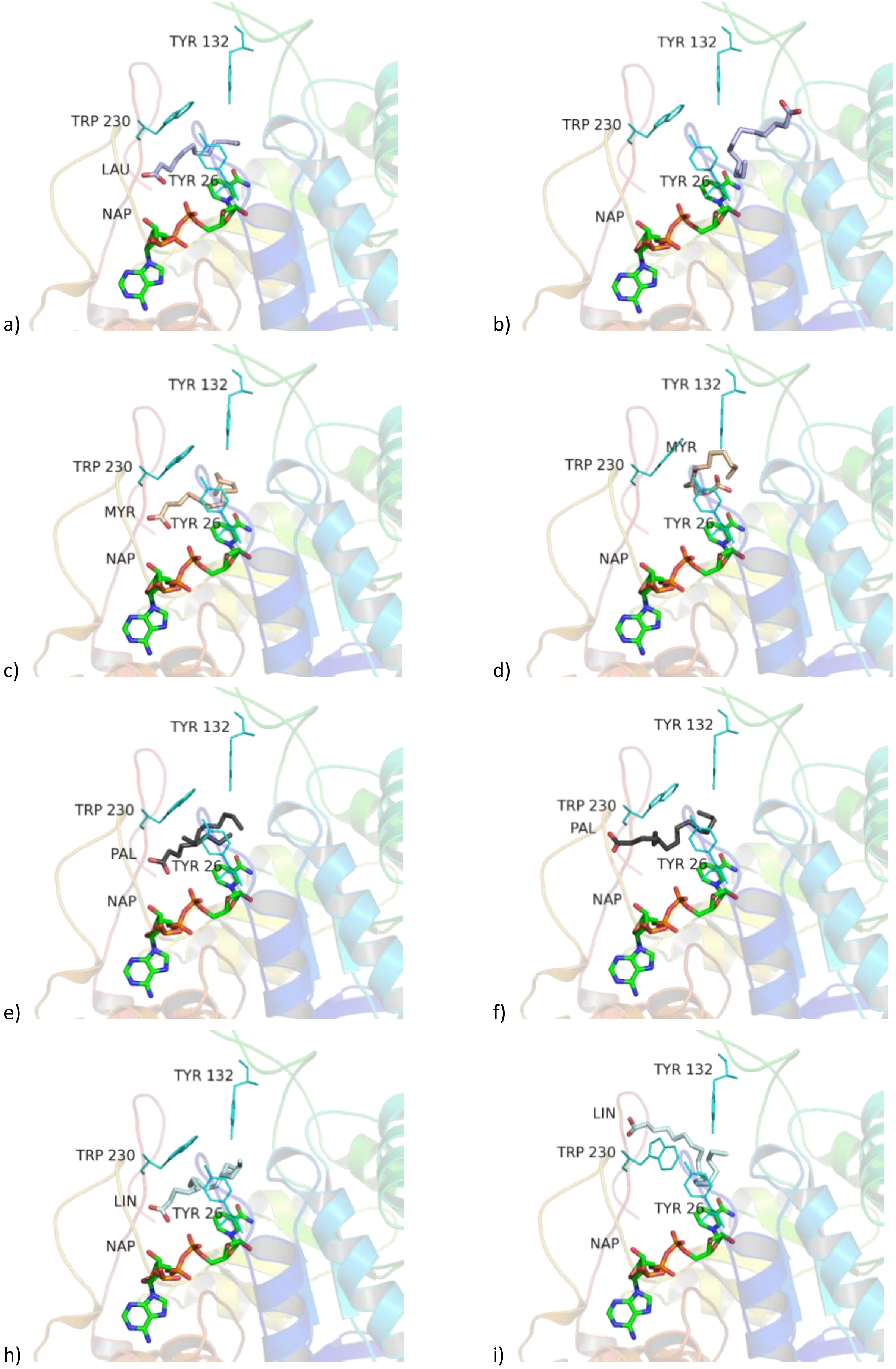

Supporting Figure S2. Lauric acid (a,b), miristic acid (c,d), palmitic acid (e,f) and linoleic acid (h,i) in the unproductive (left) and productive (right) position.

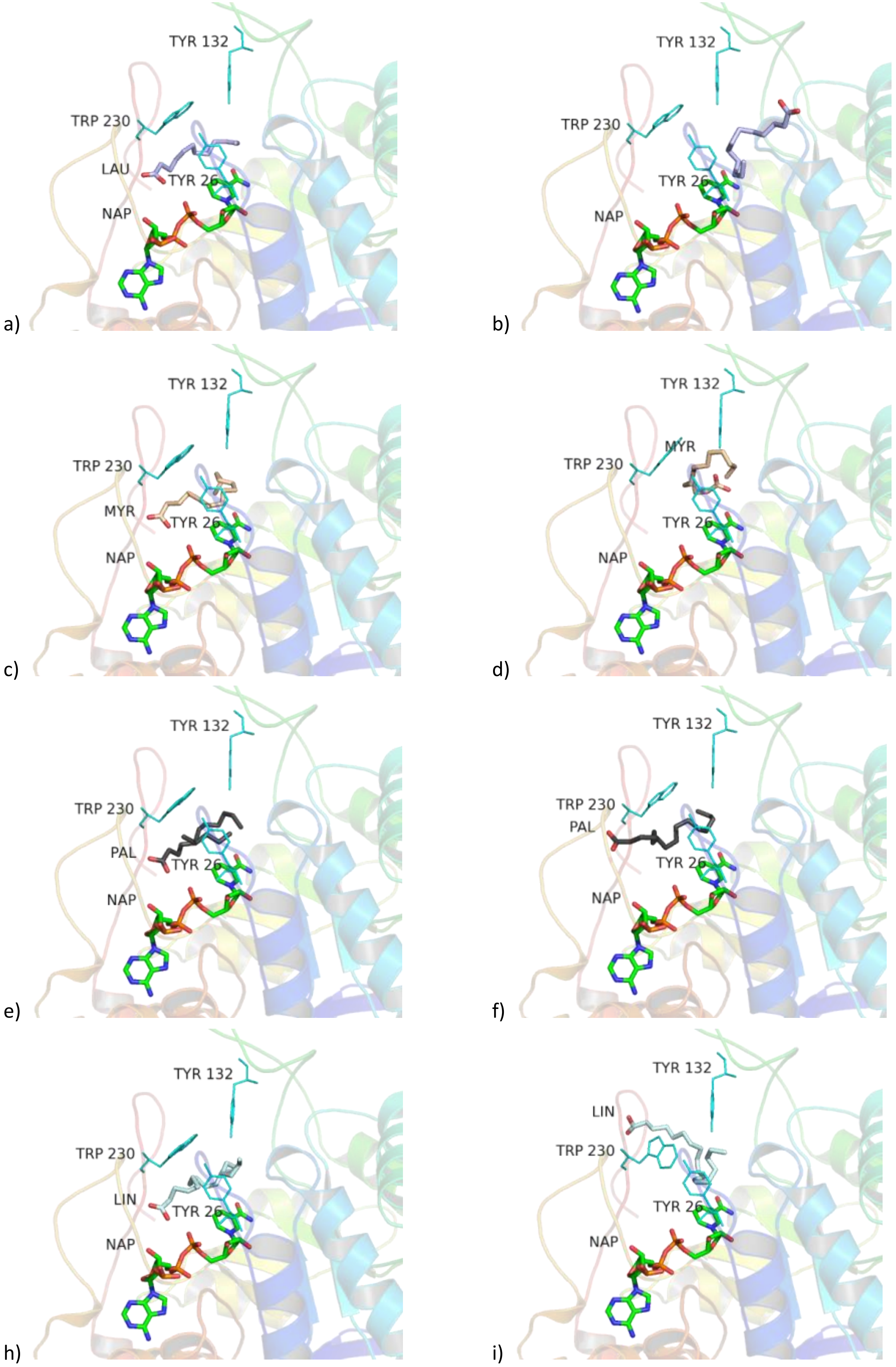

Supporting Figure S2. Lauric acid (a,b), miristic acid (c,d), palmitic acid (e,f) and linoleic acid (h,i) in the unproductive (left) and productive (right) position.
