# Supplementary material for: Hypothesis on Serenoa repens (Bartram) small extract inhibition of prostatic 5α-reductase through an in silico approach on 5β-reductase x-ray structure
Source: PeerJ. 2016 Nov 22;4:e2698. doi: 10.7717/peerj.2698 (PMC5126621; doi:10.7717/peerj.2698)
Supplement: Figure S3 — Binding site residues are sharpened in light green; active site residues are sharpened in olive green. [file peerj-04-2698-s008.pdf]

```
1    -----------------------------MATATGVAEERLLAA--LAYLQCAVGCAVFA   29  P18405   S
1    MDLSAASHRIPLSDGNSIPIIGLGTYSEPKSTPKGACATSVKVAIDTGYR-HIDGAYIYQ   59  P51857   A
                                   :* .*..   : .*   .*     *. ::

30   RNRQTNSVYGRHALPSHRL----RVPARAAWVVQELPSLALPLYQYA-------------   72  P18405   S
60   NEHEVGEAI-REKIAEGKVRREDIFYCGKLWATNHVPEMVRPTLERTLRVLQLDYVDLYI  118  P51857   A
     .:::. ..  *. : . ::     . .   *..:.:*.:. *  : :

73   ----------SESAPRLRSAPNCILLAMFLVHYGHRCLIYPF------------------  104  P18405   S
119  IEVPMAFKPGDEIYPRDEN-------GKWLYHKSNLCATWEAMEACKDAGLVKSLGVSNF  171  P51857   A
               .*  ** ..       . :* * .. *  :

105  ---------LMRGGKPM---------PLLACTMAIMFCTCNGYLQSRYLSHCAVYADDWV  146  P18405   S
172  NRRQLELILNKPGLKHKPVSNQVECHPYFTQPKLLKFCQQHDIVITAYSPLGTSRNP---  228  P51857   A
                 * *           * ::    : **  .  : : *    :

147  TDPRFLIGFGLWLTGMLINIHSDHILRNLRKPGD-------TGYKIPRGGLFEYVTAANY  199  P18405   S
229  ----------IWVNVSSPPLLKDALLNSLGKRYNKTAAQIVLRFNIQRGVVVI----P--  272  P51857   A
               :*:.     : .* :*..* *  :         ::* ** :.

200  FGEIMEWCGYALASWSVQGAAFAFFTFCFLSGRAKEHHE----------WYLRKFEEYPK  249  P18405   S
273  -------KSFNL---ERIKENFQIFDFSLTEEEMKDIEALNKNVRFVELLMWRDHPEYPF  322  P51857   A
             .: *   .     * :* *.: . . *: .              *.. ***

250  FRKIIIPFLF  259  P18405   S5A1_HUMAN
323  HDEY------  326  P51857   AK1D1_HUMAN
     . :
```

Supporting Figure S3. 5AR type 1 and 5BR sequence alignment. Binding site residues are sharpened in light green; active site residues are sharpened in olive green.
