# Supplementary material for: Hypothesis on Serenoa repens (Bartram) small extract inhibition of prostatic 5α-reductase through an in silico approach on 5β-reductase x-ray structure
Source: PeerJ. 2016 Nov 22;4:e2698. doi: 10.7717/peerj.2698 (PMC5126621; doi:10.7717/peerj.2698)
Supplement: Figure S4 — Binding site residues are sharpened in light green; active site residues are sharpened in olive green. [file peerj-04-2698-s009.pdf]

```
1     MQV--QCQQSPVLAGS-ATLVALGALALYV-------------AKPSGYGKHTES-----   39  P31213   S
1     MDLSAASHRIPLSDGNSIPIIGLGTYSEPKSTPKGACATSVKVAIDTGYRHIDGAYIYQN   60  P51857   A
      *::   .:: *:  *.   ::.**: :                *  :** :   :

40    -------LK--------PAATRLPARAAWFLQELPSFAVPA-------------------   65  P31213   S
61    EHEVGEAIREKIAEGKVRREDIFYCGKLWATNHVPEMVRPTLERTLRVLQLDYVDLYIIE  120  P51857   A
             ::             : .   *  :.:*.:. *:

66    ----------------------------------------GILARQPLS---------LF   76  P31213   S
121   VPMAFKPGDEIYPRDENGKWLYHKSNLCATWEAMEACKDAGLVKSLGVSNFNRRQLELIL  180  P51857   A
                                              *::    :*         ::

77    GPPGT----VLLGLFCVHYFHRTFVYSLLNRGRPYPAILILRGTAFCTGNGVLQGYYLIY  132  P31213   S
181   NKPGLKHKPVSNQVECHPYFTQPK------------------LLKFCQQHDIVITAYSPL  222  P51857   A
        **     *   : *  ** :                       **  . ::   *

133   CAEYPDGWYTDIRFSL--GVFLFILGMGINIHSDYILRQLRKPGEISYRIPQGGLFTYVS  190  P31213   S
223   GTSRNPIWVNVSSPPLLKDALLNSLGKRYNKTAAQIV--------LRFNIQRGVV-VIPK  273  P51857   A
       :.    * .     *   .:*  **   *  :  *:        : :.* :* : .  .

191   GAN--FLGEIIEWIGYALATWSLPALAFAFFSLCFLGLRAFHHHRFYLKMFEDYPKSRKA  248  P31213   S
274   SFNLERIKENFQIFDFSLTEEEMKDIEALNKNVRFVELLMWRDHP-------EYPFHDEY  326  P51857   A
      . *   : * :: : ::*:  .:  :     .: *: *  ::.*        :**   :

249   LIPFIF  254   P31213   S5A2_HUMAN
327   ------  326   P51857   AK1D1_HUMAN
```

Supporting Figure S4. 5AR type 2 and 5BR sequence alignment. Binding site residues are sharpened in light green; active site residues are sharpened in olive green.
