# Supplementary figures and images for: Hypothesis on Serenoa repens (Bartram) small extract inhibition of prostatic 5α-reductase through an in silico approach on 5β-reductase x-ray structure
Source: PeerJ. 2016 Nov 22;4:e2698. doi: 10.7717/peerj.2698 (PMC5126621; doi:10.7717/peerj.2698)

DUD-E 1

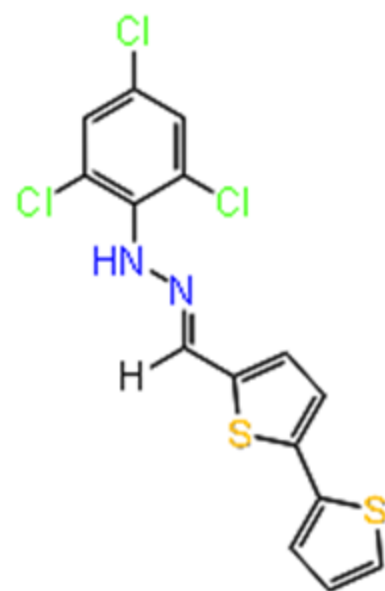

DUD-E 2

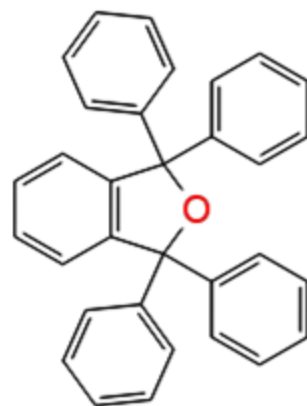

DUD-E 3

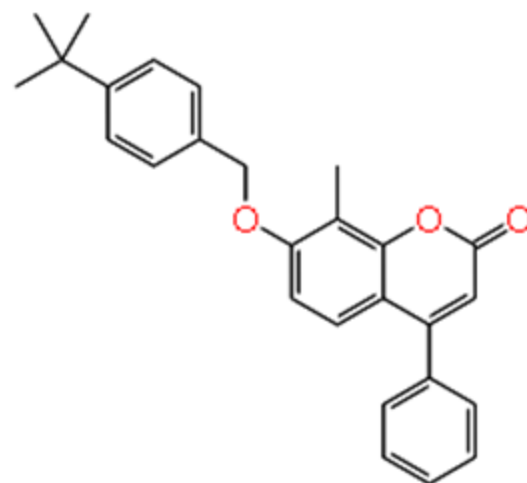

DUD-E 4

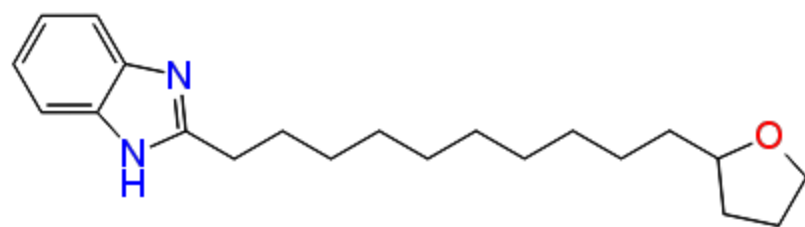

DUD-E 5

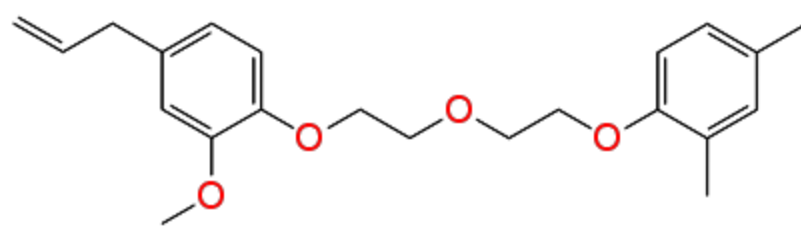

DUD-E 6

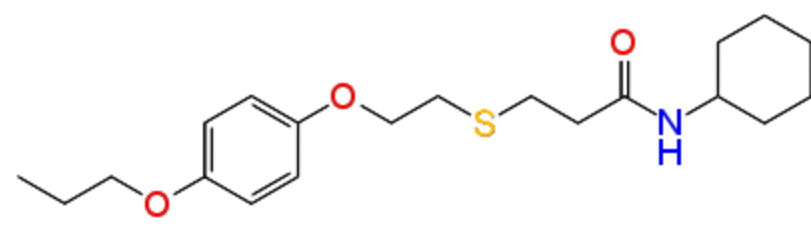

Supporting Figure S5. Formulas of the top 6 decoys generated by DUD-E.

Supplement: Figure S5 [file peerj-04-2698-s010.pdf]
